# Chikungunya in U.S. Travelers: A Double Challenge

**DOI:** 10.4269/ajtmh.18-0170

**Published:** 2018-07

**Authors:** Fabrice Simon, Anne-Laurence Demoux

**Affiliations:** Laveran Military Teaching Hospital; Marseille, France; E-mail: simon-f@wanadoo.fr; Nord University Teaching Hospital; Marseille, France; E-mail: annelaurence.baiada@ah-hm.fr

Dear Sir,

We read with great interest the recent article by Lindsey et al.^1^ on the epidemiological and clinical profile of roughly four thousand U.S. travelers infected with chikungunya (CHIK) virus in 2014–2016, associated with a huge outbreak in the Caribbean region.The authors clearly show the real risk of introduction of this virus into the United States, as at least one quarter of the travelers were viremic, and most cases were observed in warm months corresponding to the highest activity of *Aedes* mosquito vectors. There is no doubt that this threat will persist in the coming years, even if the CHIK epidemic in the Americas decreases. Demonstration that this danger persists is that both southern France and Brazil underwent autochthonous transmission of CHIK virus after an unexpected introduction of a strain from Africa,^2–4^ rather than from the Americas. As Lindsey et al. wrote, preparedness must be pursued and reinforced to better detect new imported cases and limit autochthonous transmission in the United States.

As physicians, we also would like to warn about the second big challenge for North America in relation to CHIK infection in U.S. travelers: the high risk of inappropriate case management of patients with chronic post-CHIK disorders. More than the half of the CHIK-infected U.S. travelers presented at least one risk factor for long-lasting rheumatic and/or general symptoms: female gender and age greater than 40 years. In2deed, post-CHIK syndromes are unknown clinical entities for most physicians in non-epidemic areas. Fortunately, after huge outbreaks in the Indian Ocean and the Americas, experience-based guidelines^5,6^ are now available online to help physicians treat patients with this disabling disease. The strategy is based on an attentive physical examination to set up a precise diagnosis, drugs against pain and articular inflammation, and physiotherapy against stiffness and muscle loss. Any patient with symptoms refractory to this first-line treatment should be referred to a rheumatologist to consider the diagnosis of post-CHIK chronic inflammatory arthritis, which may develop in a very small percentage of patients. Thus, beside the public health strategy of vector control and early case recognition, the U.S. response against CHIK should also include a network to adequately manage CHIK-infected travelers, to avoid inappropriate management and despair, as commonly observed in other non-epidemic countries.
